# Sectional Anatomy Quiz – VIII

**DOI:** 10.22038/AOJNMB.2022.55234.1383

**Published:** 2022

**Authors:** Geoffrey T Murphy, Muhammad Azaan Khan, Rashid Hashmi

**Affiliations:** Rural Medical School, University of New South Wales (UNSW), Wagga Wagga, NSW, Australia

**Keywords:** Anatomy, Brain, Basal Ganglia

## Abstract

This series lists a pictorial quiz pertaining to identification of normal and abnormal anatomical structures and landmarks at a given level on computed tomography (CT). Readers are expected to identify and appreciate the changes from normal anatomy and variations of a given pathology. It is anticipated that this series will enhance the understanding of sectional anatomy of the brain to aid in brain CT interpretation.

**Figure 1:**
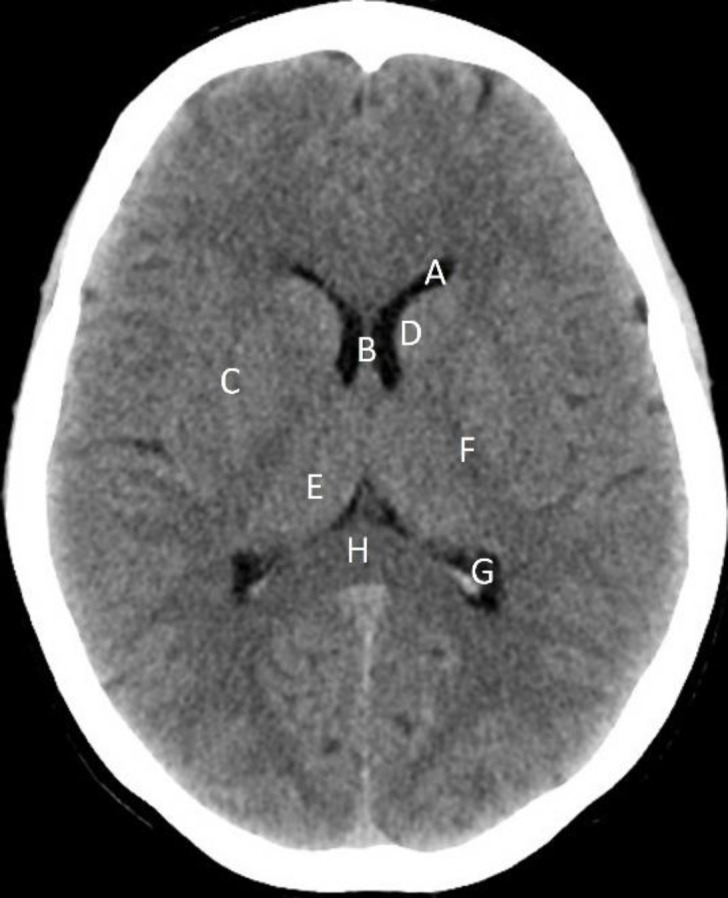
Identify the normal anatomical structures labelled **A** to **H** on a non-contrast axial CT brain of a 43 years-old male

**Answers**

A: Frontal (anterior) horn of left lateral ventricle

B Septum pellucidum

C: Right lentiform nucleus

D: Head of left caudate nucleus

E: Right thalamus

F: Posterior limb of the left internal capsule 

G: Occipital (posterior) horn of the left lateral ventricle

H: Splenium of corpus callosum 

 The image is at the level of level of the basal ganglia which is group of paired deep grey matter nuclei including caudate and lentiform nucleus. Thin linear high density seen in the occipital horns of the lateral ventricles is due to calcification of the choroid plexus.

**Figure 2:**
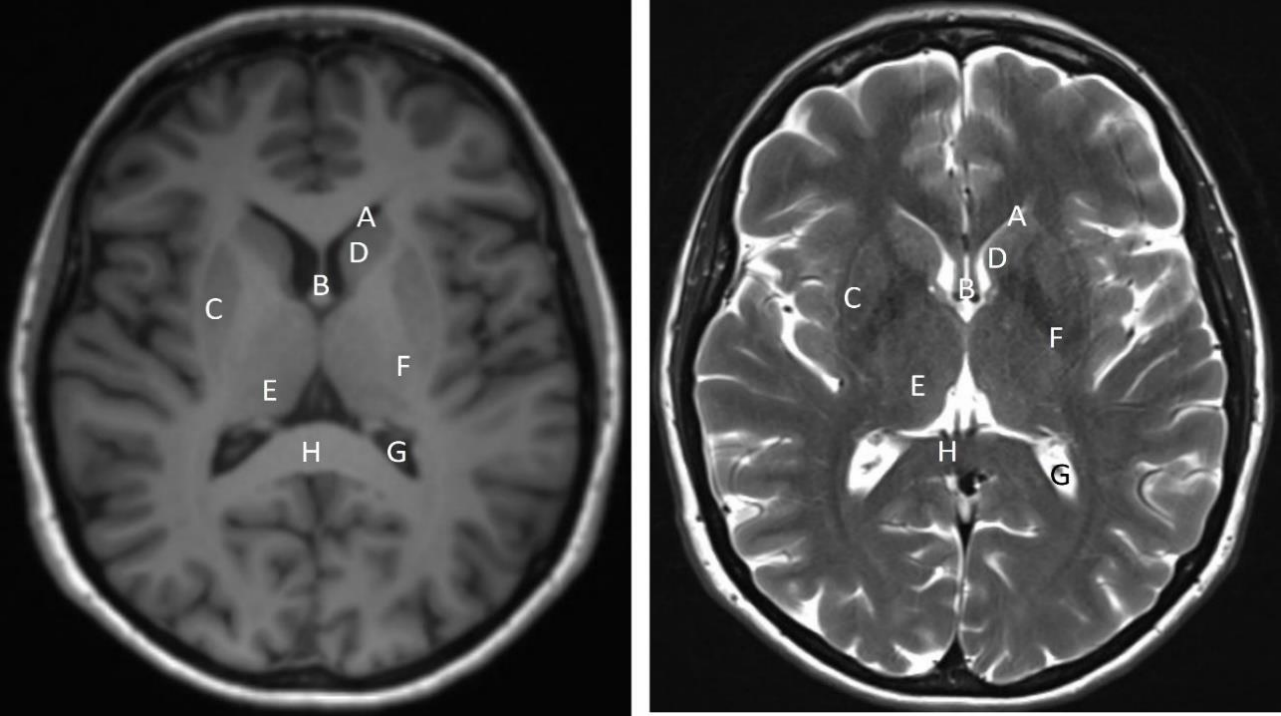
T1 (left) and T2 (right) weighted axial MR images of the brain of a 45 years-old female at the level of the basal ganglia shows normal anatomy. Cerebrospinal fluid (and fluid elsewhere in body) appears hypointense (black) on T1 and hyperintense (white) on T2 weighted images respectively. Fat appears hyperitense on both T1 and T2 weighted images. It is to be noted that signal intensity of a given structure changes on various sequences used during MR imaging

Comparison with Figure 1 will help appreciating difference in appearance of normal structures on MR and CT images (**A**-**H**)

**Figure 3:**
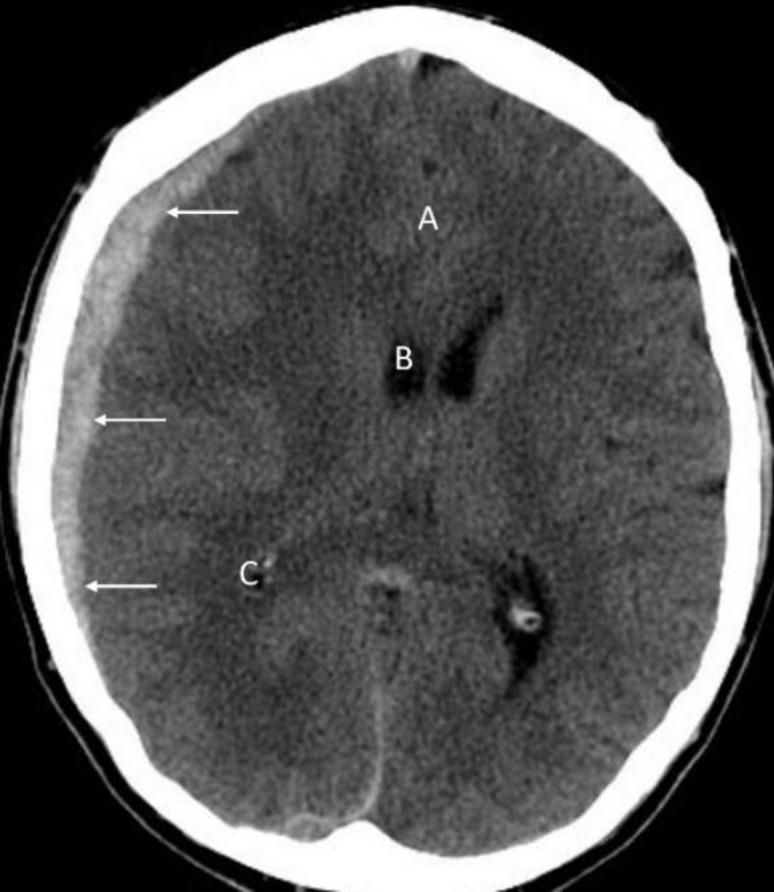
Non-contrast axial CT brain of a 31 years-old male with an acute subdural hematoma (**arrows**) following a road traffic accident. Note the midline shift (**A**) and compression of the frontal (**B**) and occipital (**C**) horns of the lateral ventricle suggesting mass effect

 Acute subdural hematoma characteristically appears as a crescent shaped high density area.

Sub-acute subdural hematoma can be isodense to the normal brain parenchyma makings its 

detection difficult on CT. Chronic subdural hematoma is lower in density compared to the brain parenchyma.

**Figure 4:**
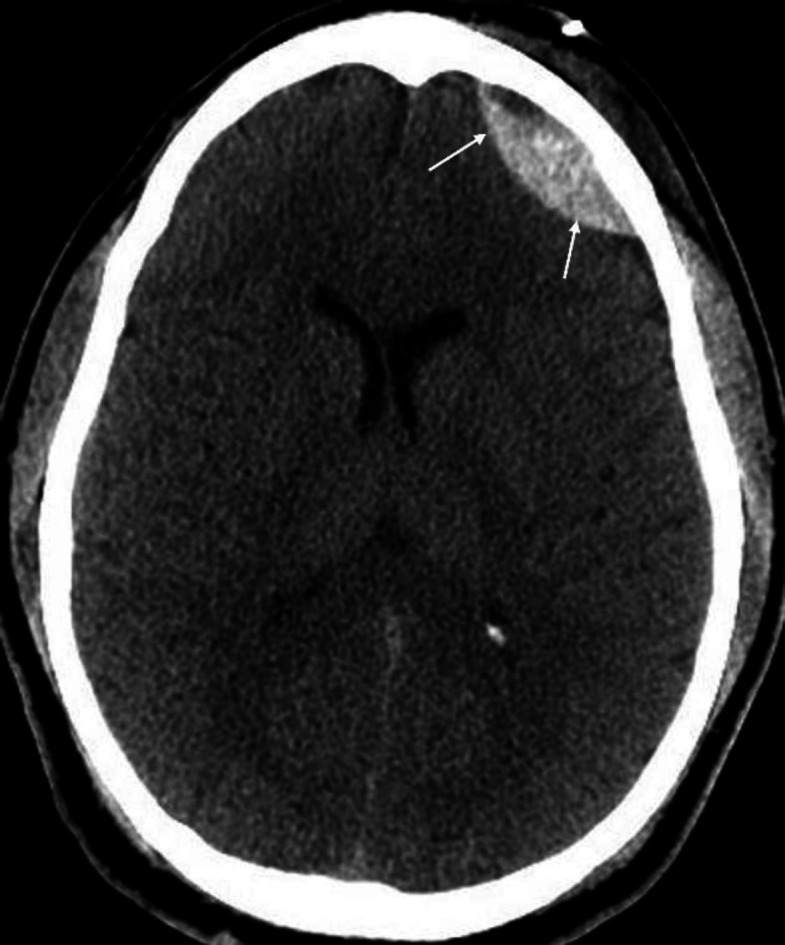
Non-contrast axial CT brain of a 31 years-old male with an epidural (extra-dural) hematoma (**arrows**) shows a bi-convex hyperdense area in the left frontal region. Typically, an epidural hematoma is lentiform (bi-convex, lens shaped, lemon shaped etc.) and does not cross the sutures. In comparison, a subdural hematoma is cresenteric (moon shaped, sickle shaped, banana shaped etc.) and can cross the suture or midline

**Figure 5:**
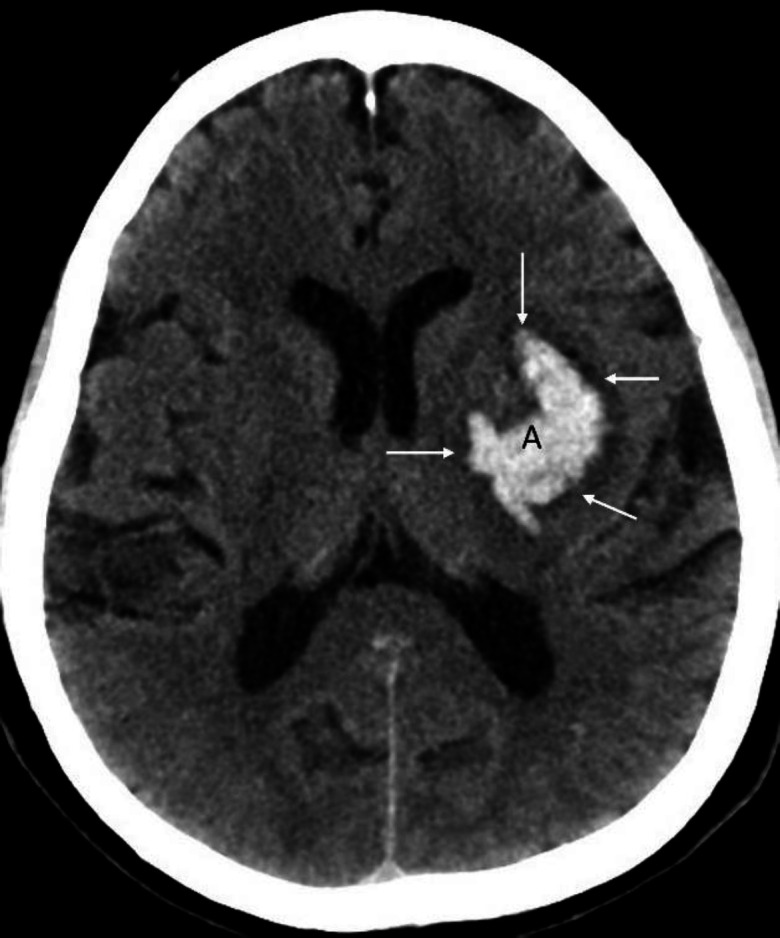
Non-contrast axial CT brain of a 79 years-old male with uncontrolled hypertension shows an intracerebral hemorrhage (**A**). Hemorrhage is centered over the left sided lentiform nucleus. Low density area surrounding the hemorrhage (**arrows**) represent peri-focal oedema. No significant mass effect on surrounding structures is noted on this image

**Figure 6:**
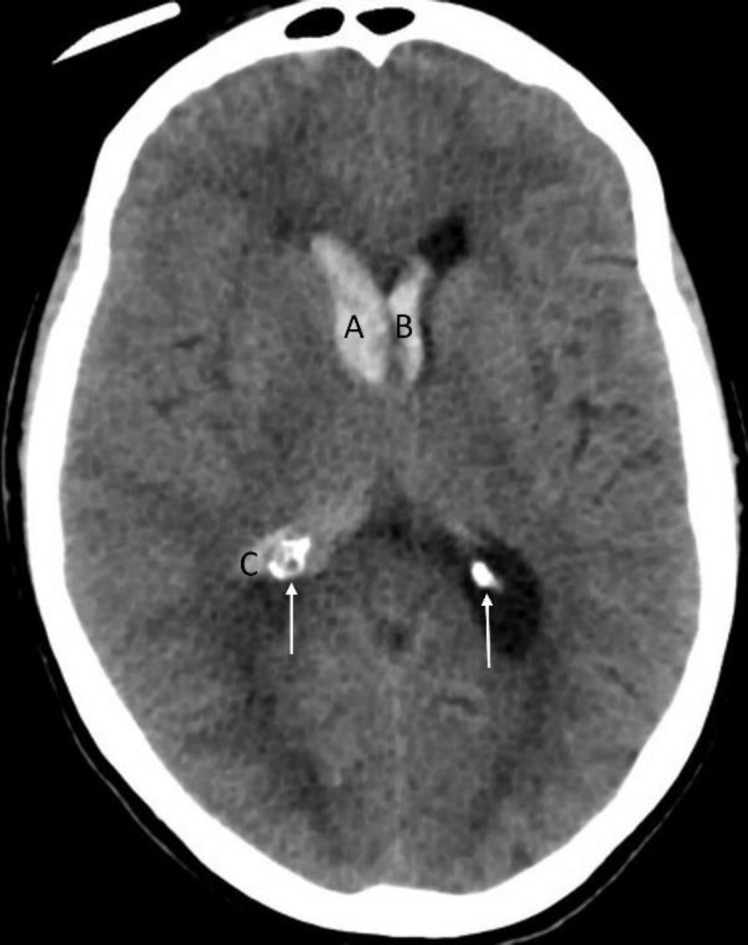
Non-contrast axial CT brain of a 78 years-old female with an intracerebral bleed showing intraventricular extension of bleed in the lateral ventricles (**A**-**C**). Note difference in the density of an acute bleed with that of calcification (**arrows**) of the choroid plexus. It is important to appreciate that, unlike acute bleed, density of calcification parallels that of the calvarium

**Figure 7:**
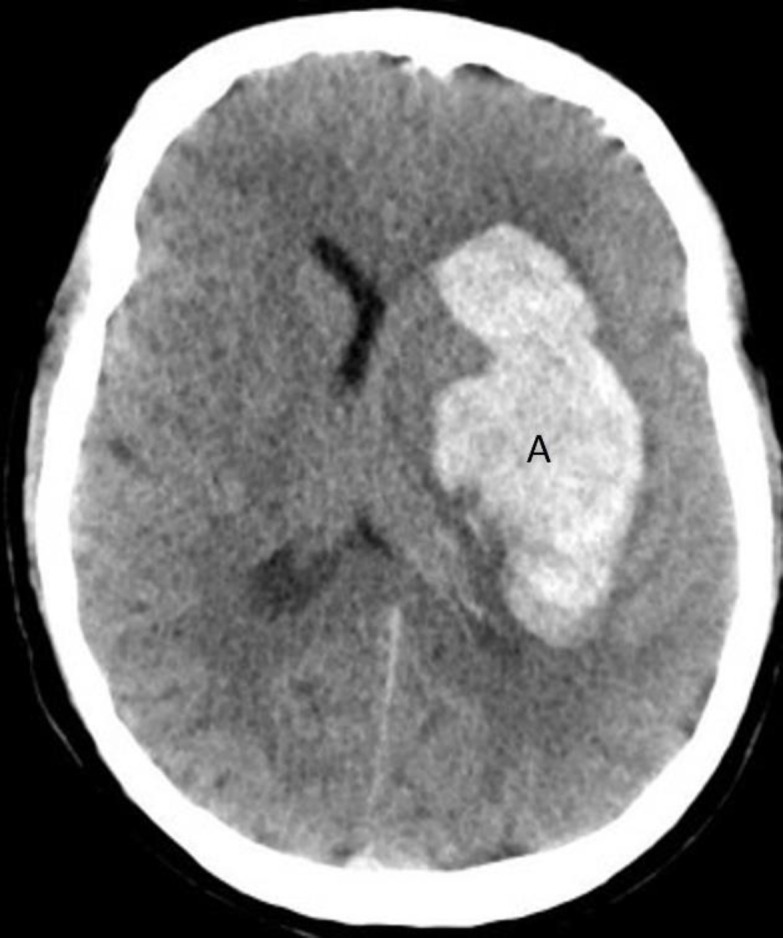
Non-contrast axial CT brain of a 51 years-old female with uncontrolled hypertension shows an intracerebral hemorrhage involving the left basal ganglia (**A**). Note the compression of the ipsilateral ventricle and midline shift

**Figure 8:**
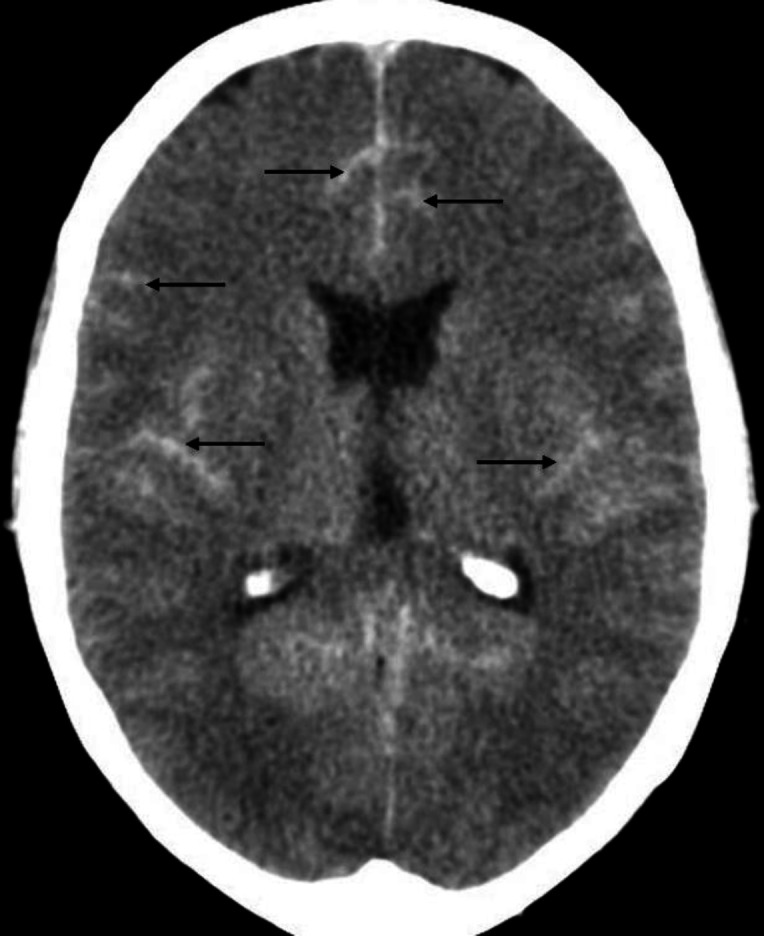
Non-contrast axial brain of a 72 years-old female with a subarachnoid hemorrhage shows linear high density areas involving the cerebral sulci (**arrows**). Note prominent calcification of the choroid plexus in the occipital horns of the lateral ventricle bilaterally

**Figure 9:**
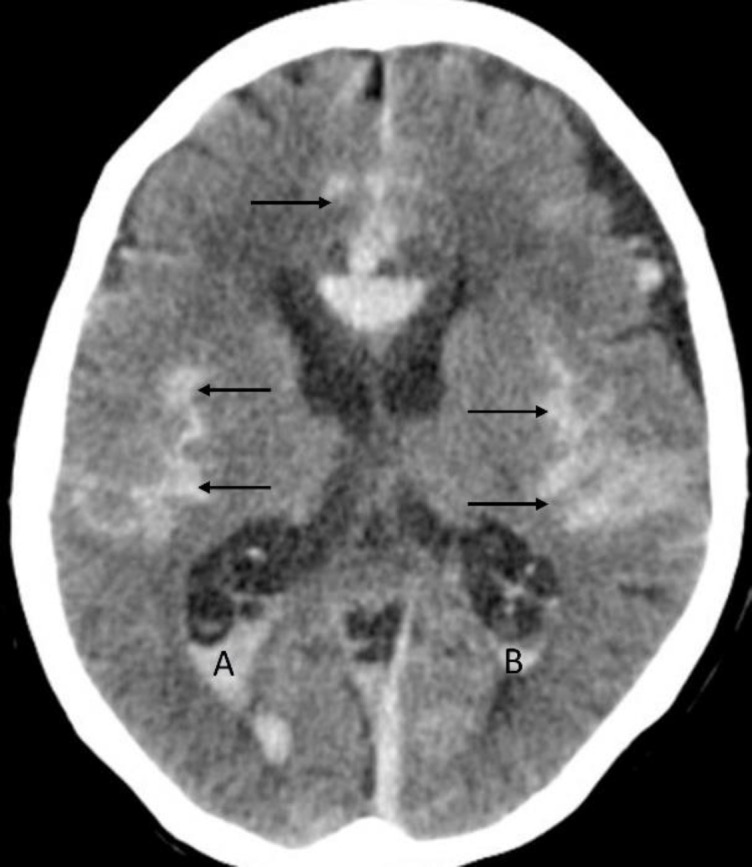
Non-contrast axial CT brain of a 67 years-old female with subarachnoid hemorrhage (**arrows**) and intraventricular extension in the occipital horns of the lateral ventricle bilaterally (**A**-**B**)

**Figure 10:**
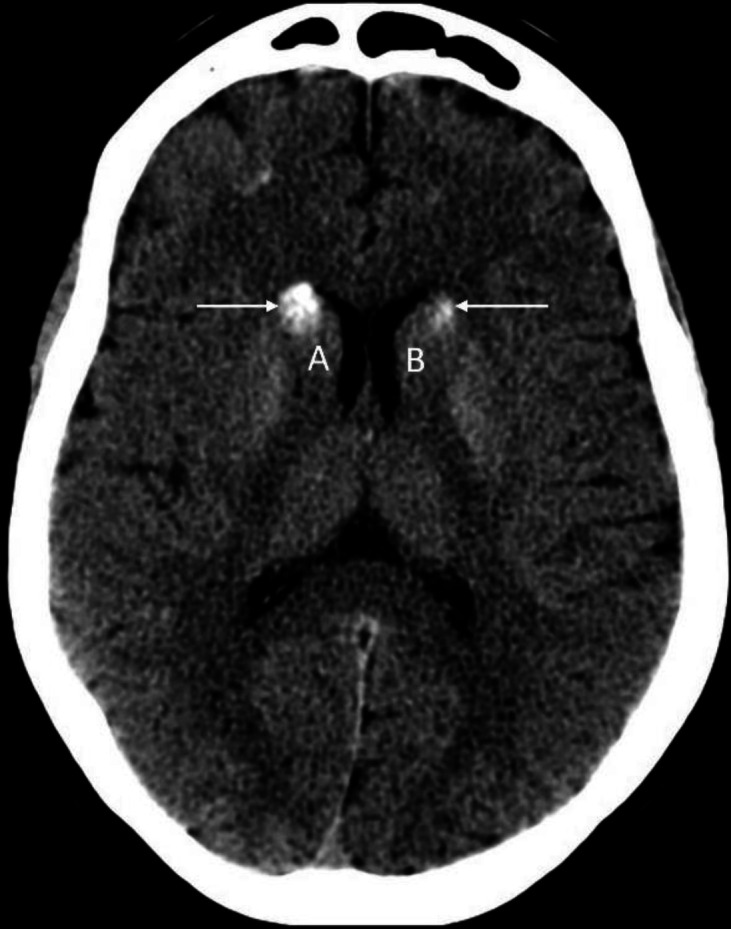
Non-contrast axial CT brain of a 56 years-old male shows calcification (**arrows**) of the head of caudate nucleus bilaterally (**A**-**B**). Calcification in this location and other part of basal ganglia can be seen incidentally in older individuals and generally considered to be of no clinical significance. However, entities like Fahr disease, lead and carbon monoxide poisoning, tuberculosis, neuro-cysticercosis, toxoplasmosis and some metabolic disorders (e.g. hypo and hyperparathyroidism etc.) can also result in calcification

**Figure 11:**
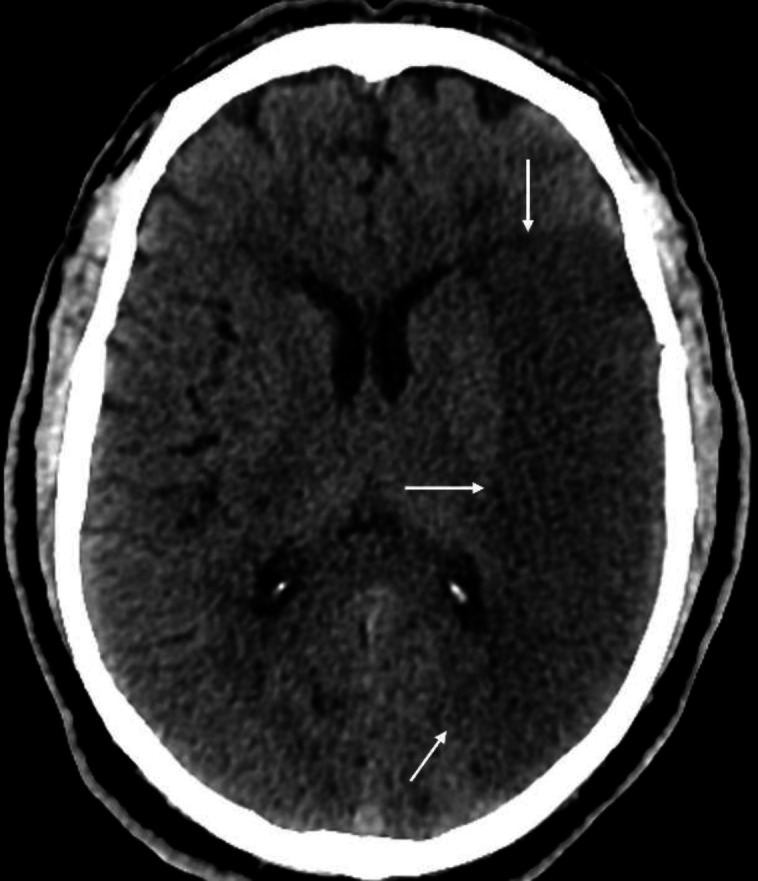
Non-contrast axial CT brain of a 55 years-old female with acute ischemic infarction involving the left middle cerebral artery territory. Patient presented with right hemiplegia that evolved over past 9 hours. Large ill-defined low density area (**arrows**) involving the left parietal lobe and causing effacement of the adjacent sulci is the characteristic finding of an acute ischemic infarction on non-contrast CT. Note absence of any significant mass effect

**Figure 12:**
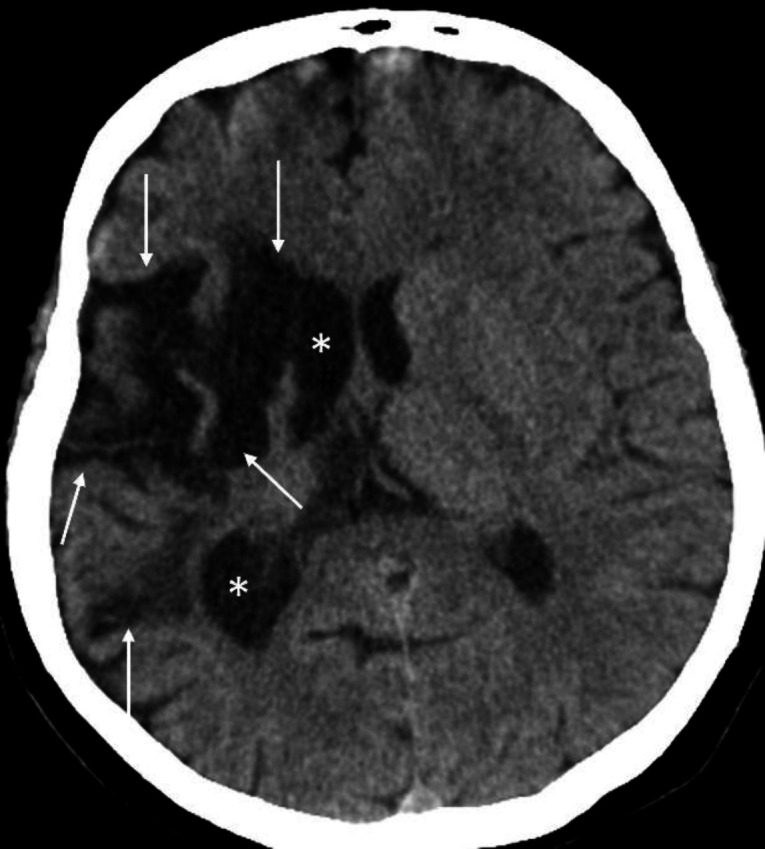
Non-contrast axial CT brain of an 82 years-old female with a history of a prior stroke shows an old infarction in the right middle cerebral artery territory (**arrows**). Note the dilatation of the ipsilateral ventricle (**asterisks**) due to loss of brain volume

## Recommendations for Further Readings

1. Ryan S, McNicholas M, Eustace SJ. Anatomy for diagnostic imaging e-book. 3^rd^ Ed. New York: Elsevier Health Sciences; 2011.

2. Currie S, Kennish S, Flood K. Essential Radiological Anatomy for the MRCS. Cambridge University Press. 2009.

3. Moeller T, Reif E. Pocket atlas of sectional anatomy. Computed tomography and magnetic resonance imaging. Vol. 1 Head and neck. Stuttgart: Thieme; 2013.

